# Ammonia Storage in Metal–Organic Framework Materials: Recent Developments in Design and Characterization

**DOI:** 10.1021/accountsmr.4c00183

**Published:** 2024-10-04

**Authors:** Wanpeng Lu, Dukula De Alwis Jayasinghe, Martin Schröder, Sihai Yang

**Affiliations:** † Department of Chemistry, University of Manchester, Manchester, M13 9PL, U.K.; ‡ College of Chemistry and Molecular Engineering, Beijing National Laboratory for Molecular Sciences, Peking University, Beijing, China, 100871

## Abstract

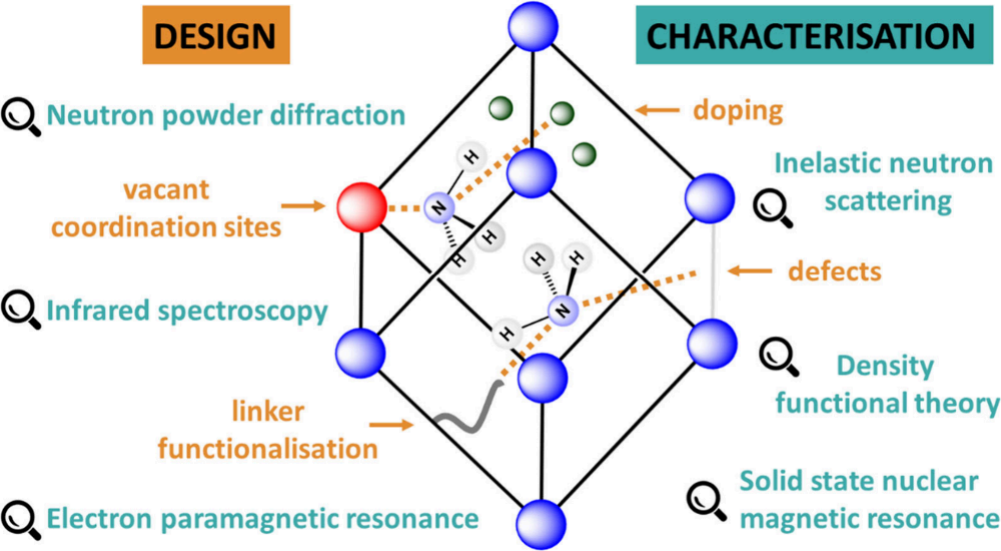

Since the advent of the Haber–Bosch process in 1910, the global demand for ammonia (NH_3_) has surged, driven by its applications in agriculture, pharmaceuticals, and energy. Current methods of NH_3_ storage, including high-pressure storage and transportation, present significant challenges due to their corrosive and toxic nature. Consequently, research has turned towards metal–organic framework (MOF) materials as potential candidates for NH_3_ storage due to their potential high adsorption capacities and structural tunability. MOFs are coordination networks composed of metal nodes and organic linkers, offering unprecedented porosity and surface area, and allowing incorporation of various functional groups and metal sites that can enhance NH_3_ adsorption. However, the stability of MOFs in the presence of NH_3_ is a significant concern since many degrade upon exposure to NH_3_, primarily due to ligand displacement and framework collapse. To address this, recent studies have focused on the synthesis and postsynthetic modification of MOFs to enhance both NH_3_ uptake and stability. In this Account, we summarize recent developments in the design and characterization of MOFs for NH_3_ storage. The choice of metal centers in MOFs is crucial for stability and performance. High-valence metals such as Al^III^ and Ti^IV^ form strong metal–linker bonds, enhancing the stability of the framework to NH_3_. The MFM-300 series of materials composed of high-valence 3+ and 4+ metal ions and carboxylic linkers demonstrates high stability and high NH_3_ uptake capacities. Ligand functionalization is another effective strategy for improving the NH_3_ adsorption. Polar functional groups such as –NH_2_, –OH, and –COOH enhance the interaction between the framework and NH_3_, particularly at low partial pressures, while postsynthetic modification allows fine-tuning of these functionalities to optimize the framework for higher adsorption capacities and stability. For example, MFM-303(Al), incorporating free carboxylic acid groups, exhibits a high NH_3_ packing density comparable to that of solid NH_3_. Creating defect sites by removing linkers or adding metal ions increases the number of active sites available for NH_3_ adsorption and shows promise for enhancing uptake. UiO-66, a stable MOF framework, can be modified to include defect sites, significantly enhancing the level of NH_3_ uptake. The full characterization of MOFs and especially their interactions with NH_3_ are vital for understanding and improving their performance. Techniques such as neutron powder diffraction (NPD), inelastic neutron scattering (INS), diffuse reflectance infrared Fourier transform spectroscopy (DRIFTS), electron paramagnetic resonance (EPR) spectroscopy, and solid-state nuclear magnetic resonance (ssNMR) spectroscopy can elucidate host–guest interactions and binding dynamics between NH_3_ and the framework structure and afford crucial information for the future design and rational development of new sorbents. This Account highlights our current strategies for the synthesis and characterization of MOFs for NH_3_ capture, providing an overview of this rapidly evolving field.

## 1 Introduction

Since the development of the Haber–Bosch process in 1910, the demand for NH_3_ has increased significantly, particularly due to applications in agriculture, drug development, and energy production.^1,2^ According to the Natural Mineral Commodity Summaries, the past 10 years have witnessed a 9.5% increase in total global annual NH_3_ production, reaching ca. 150 million tons in 2022.^3^ Other reports have demonstrated production of up to 236 million tons per annum, with a 22% increase in production demand expected by 2030.^4,5^ NH_3_ has a high hydrogen (H_2_) content (17.8 wt%) compared with other carriers such as HCOOH and CH_3_OH.^6^ Therefore, NH_3_ is also of interest to the energy sector, particularly as a potential H_2_ carrier to help reach the “Net-Zero” target by 2050 as defined in “The Paris Agreement”.

NH_3_ is a highly corrosive and toxic gas under ambient conditions and is also an alkaline environmental pollutant. NH_3_ emissions through accidental leakages during transportation, from livestock farming, and from vehicles contribute to elevated levels of NH_3_ in the atmosphere, which lead to potential public health implications and economic costs. Current methods for NH_3_ capture and removal include retrieval of solid ammonium sulfate by reaction with H_2_SO_4_, biological fixation routes, and use of permeable membranes.^7^ However, these are subject to significant problems including low selectivity, high management and energy costs, and further waste production such as release of NO_*x*_ gases during the capture and treatment process. As an alternative, porous materials for physisorption and chemisorption of NH_3_ have been extensively studied in the recent years. This includes conventional porous materials, such as activated carbons, mesoporous silica, and zeolites,^8^ but new emerging functional materials including metal organic frameworks (MOFs),^9^ covalent organic frameworks (COFs),^10^ and porous polymers^11^ are of significant interest. Among these, MOFs, formed by the linking of metal centers or cluster nodes with bridging organic ligands,^14^ show promising performance for NH_3_ storage with high potential uptake capacities. This is typically achieved by the presence of vacant coordination sites at the metal or cluster center and/or by interaction with acidic and other functional groups on the organic linker leading to hydrogen bonded networks within the pores. However, due to the corrosive nature of NH_3_, host material degradation is common and is a major challenge for the development of new adsorbents that are sufficiently robust over multiple adsorption–desorption cycles. Many MOFs degrade upon contact with NH_3_ or after only a few adsorption–desorption cycles, and thus, this area is really in its infancy in terms of the design and synthesis of porous materials that could function in real-world applications. But, more recently, MOFs have been reported that show high stability under extreme conditions including in strong alkaline solutions.

Though reviews on NH_3_ capture within porous materials have been published,^12,13^ we give here a systematic summary of our strategies to develop MOFs with high stability and capacity. We review for the first time the host–guest supramolecular interactions between NH_3_ and the pores within the MOFs. This is crucial for a fuller understanding of the mechanisms for NH_3_ uptake and the design and development of improved systems in the future. In addition, state-of-the-art diffraction, scattering, and spectroscopic methods for studying and defining host–guest binding interactions of MOFs with NH_3_ are illustrated and discussed to give important insights into the design and characterization of porous robust MOFs for NH_3_ capture.

## 2 Ammonia Adsorption in MOFs: A General View

MOFs are coordination networks of mono- or polynuclear metal coordination nodes linked by organic bridging ligands.^14^ Combinations of different complex nodes and organic linkers have led to a very significant growth of MOF materials reported over the past two decades.^15^ Careful selection of organic and inorganic components can yield highly crystalline MOFs with specific desired properties, such as high porosity, high stability, or targeted pore functionality. Postsynthetic modification of MOFs is also a powerful approach to alter and tune pore functionality and reactivity. With their unprecedented porosity, MOFs have thus received increasing attention as a promising candidate for applications in a wide variety of gas separation and gas storage, including for NH_3_ capture, storage, and transport.^16^ Several approaches have been investigated including the formation of MOF composites with materials such as graphite oxide,^17^ graphene,^18^ or metal hydroxides.^19^ In general, the resultant materials show limited adsorption capacities, and the complexity of these composites has hindered further investigation of the binding affinities and structure. More recently, we have reported new MOF materials showing high NH_3_ storage capacities and stability, and these are the focus of this Account.^20−37^

The adsorption performance of any given MOF can be defined gravimetrically or volumetrically *via* the measurement of a pure or mixed component isotherm or in dynamic breakthrough experiments using NH_3_ diluted in a carrier gas. Gravimetric uptakes derived from adsorption isotherms are the most directly comparable, and it is these data that are generally provided in literature reports. It is worth noting that most MOFs reported so far for NH_3_ capture tend to be microporous and thus possess relatively narrow pores and channels. This also tends to enhance the stability of the porous material itself due to the enhanced rigidity of the framework. The uptake and binding of NH_3_ is highly affected by host–guest interactions, particularly at low partial pressures, and this encourages the design of MOFs that incorporate surface properties and functional groups that enhance and control substrate binding.

**Figure 1:**
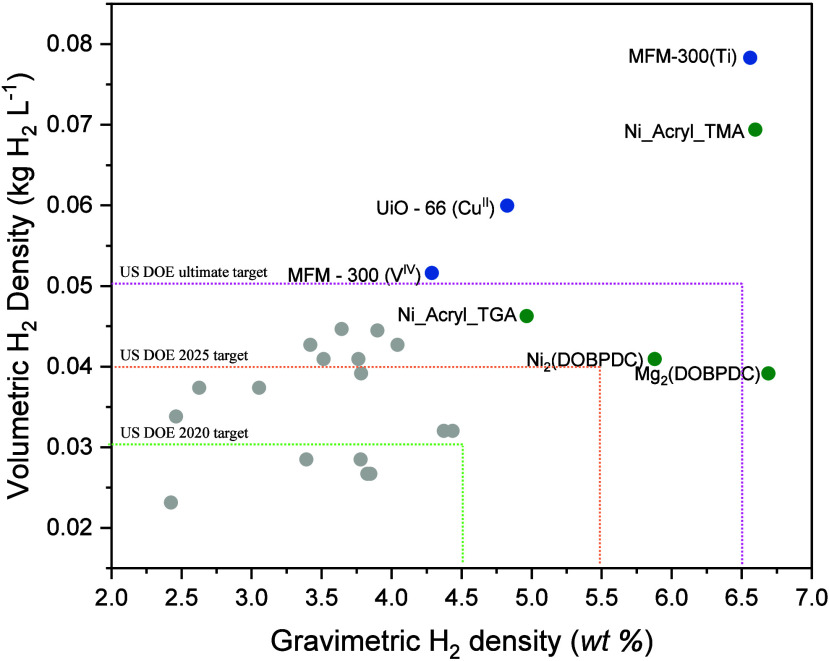
Theoretical volumetric and gravimetric density of H_2_ in state-of-the-art NH_3_-loaded MOFs. Those exhibiting high volumetric and/or gravimetric H_2_ densities are labeled; others are represented in gray.

The packing density of NH_3_ molecules in MOFs is generally similar to that of NH_3_ in the liquid state (0.69 g cm^–3^ at 240 K) and, in some cases, even close to that of solid NH_3_ (0.82 g cm^–3^ at 193 K), which makes MOFs efficient candidates for NH_3_ transportation and storage. It should be noted that various methods have been used to calculate the packing density from experimental data, and there are no commonly agreed criteria for determining this value. Pore volumes can be determined by analysis of voids within determined crystal structures, from gas sorption measurements, or from computational calculations. Volumetric density of NH_3_ within a MOF is another important parameter that needs to be considered, as the sorbate density per unit volume is an important parameter considered in the construction of storage plants.^38^Table 1 summarizes data for state-of-the-art MOFs for NH_3_ adsorption with packing and storage densities calculated from crystal structures. The latter are independent of the analyzing conditions and provide a more rigorous estimation of the pore volume. The conversion of NH_3_ to H_2_ is also of significant interest, and more efficient conversion methods need to be developed. Formally NH_3_-loaded MOFs have already surpassed the target goal established by the U.S. Department of Energy (DoE) (Figure 1), which defines 6.5 wt % and 0.50 kg H_2_ L^–1^, respectively, for gravimetric and volumetric energy densities.^39^

**Table 1:** Summary of NH_3_ Adsorption in Selected State-of-the-Art MOFs (uptakes are recorded at 298 K unless noted otherwise)^a^

name	uptake (mmol g^–1^)	packing density (g cm^–3^)	storage density (g cm^–3^)	notes	ref
MFM-300(V^III^)	16.1^b^	0.54	0.29	bridging Brønsted acidic hydroxyl groups; crystallinity retained after 20 cycles	(20), (21)
MFM-300(Al)	15.7^b^	0.72	0.28		
MFM-300(Fe)	15.6^b^	0.60	0.30		
MFM-300(Cr)	14.0^b^	0.51	0.27		
MFM-300(Sc)	19.5^b^	0.59	0.33	bridging Brønsted acidic hydroxyl groups; crystallinity retained after 90 cycles	(22)
MFM-300(V^IV^)	17.3^b^	0.61	0.34	bridging {μ_2_-O} groups; crystallinity retained after 20 cycles	(20)
MFM-300(Ti)	23.4^b^	0.84	0.44	bridging {μ_2_-O} groups; crystallinity retained after 25 cycles	(23)
MFM-303(Al)	8.99	0.80	0.21	*in situ* synthesized acidic linker functionalities; bridging Brønsted acidic hydroxyl groups	(24)
LiCl@MIL-53-(OH)_2_	33.9	–	–	43.4 wt% LiCl anchored within the MOF	(25)
[Mg_2_(DOBPDC)]	23.9	0.25	0.22	vacant metal sites; reversible for 3 cycles	(26)
[Ni_2_(DOBPDC)]	20.8	0.31	0.23	vacant metal sites; reversible for 3 cycles	(26)
Ni_acryl_TMA	23.5	0.70	0.39	postsynthetically modified MOF	(27)
[Cu(CYHDC)]	17.5	–	–	adsorption of NH_3_ leads to ligand displacement; packing and storage densities are not calculated, recyclable at least 6 times	(28)
Ni_acryl_TGA	17.4	0.52	0.26	postsynthetically modified MOF; crystallinity retained after 5 cycles	(27)
UiO-66-Cu^II^	16.9^b^	0.74	0.34	vacant metal sites; crystallinity retained after 15 cycles	(29)
[Ni_2_Cl_2_(BTDD)]	12.5	0.18	0.16	vacant metal sites; crystallinity retained after 3 cycles	(27)
MIL-160	12.8	0.48	0.24	heteroatoms and Brønsted acidic hydroxyl groups; crystallinity retained after 16 cycles	(30)
Al-PMOF	7.67	0.21	0.20	bridging Brønsted acidic hydroxyl groups; crystallinity retained after 2 cycles	(31)
Cu@Th-BPYDC	20.6	–	–	*in situ* Cu^II^ sites incorporated into MOF; vacant metal sites; regenerated upon heating	(32)
[Co_2_Cl_2_(BBTA)]	18.0	0.61	–	vacant metal sites; uptake retained for 3 cycles	(33)
[Ni_2_Cl_2_(BBTA)]	14.7	–	–	vacant metal sites; crystallinity retained after 1 cycle	(33)
[Cu_2_Cl_2_(BBTA)]	19.8	–	–	vacant metal sites; decomposes after 1 cycle	(33)
Fe-MIL-101-SO_3_H	17.8	–	–	Brønsted acidic sites	(34)

a DOBPDC^4–^ = 4,4-dioxido-biphenyl-3,3-dicarboxylate, TMA^2–^ = thiomallate, CYHDC^2–^ = *trans*-1,4-cyclohexanedicarboxylate, BTDD^2–^ = bis(1*H*-1,2,3-triazolato[4,5-*b*],[4′,5′-*i*])dibenzo[1,4]dioxin), BPYDC^2–^ = 2,2′-bipyridine-5,5′-dicarboxylate, BBTA^2–^ = 1*H*,5*H*-benzo(1,2-*d*:4,5-*d*′)bistriazolate. Packing density = total NH_3_ uptake/pore volume. Storage density = total NH_3_ uptake × crystallographic density.

b Uptakes are recorded at 273 K.

The functionalization of MOFs can be achieved or altered through synthetic design and postsynthetic methods. The choice of substituents on the organic linker backbone and/or change of the composition of the metal coordination or cluster node can lead to a wide range of resultant materials. However, changes of metal ion, linker, and/or reaction conditions often significantly affect the formation of the MOF product, leading to unpredicted outcomes and different products. As an alternative approach, postsynthetic modification (i.e., chemical change to the MOF once formed) allows deliberate adjustment of MOF performance and properties while potentially retaining the original MOF structure, crystallinity, and general porosity.

To improve the strength of interaction or induce new interactions between framework and polar gases, one approach focuses on metal centers with vacant sites, which offer strong affinity for Lewis basic substrates (in this case NH_3_). An alternative involves introducing functional groups onto the organic linker, such as NH_2_, OH, COOH, and SO_3_H, to enable the formation of extensive hydrogen bonding with substrates and to modulate the polarity of the framework. Herein, we discuss examples applying these different approaches in MOF synthesis to achieve improvements in NH_3_ adsorption.

## 3 Synthesis of MOFs for Ammonia Adsorption

### 3.1 Metal Center: Selection and Addition

Selection of appropriate metal centers influences the uptake capacity of the resultant MOF even at low pressure or concentration, especially through the presence of vacant metal sites. However, this approach risks framework collapse as chemisorption of NH_3_ to metal centers may lead to displacement of metal–linker bonds. Even in the absence of vacant metal sites, ligand displacement can be observed in MOFs constructed of relatively weak metal–linker bonds.^40^ Finding the balance between the adsorption capacity and stability by selection of metal or metal cluster node is therefore crucial; nonetheless, it should be noted that the metal center does not always explicitly determine the stability and NH_3_ capacity of a MOF.

**Figure 2:**
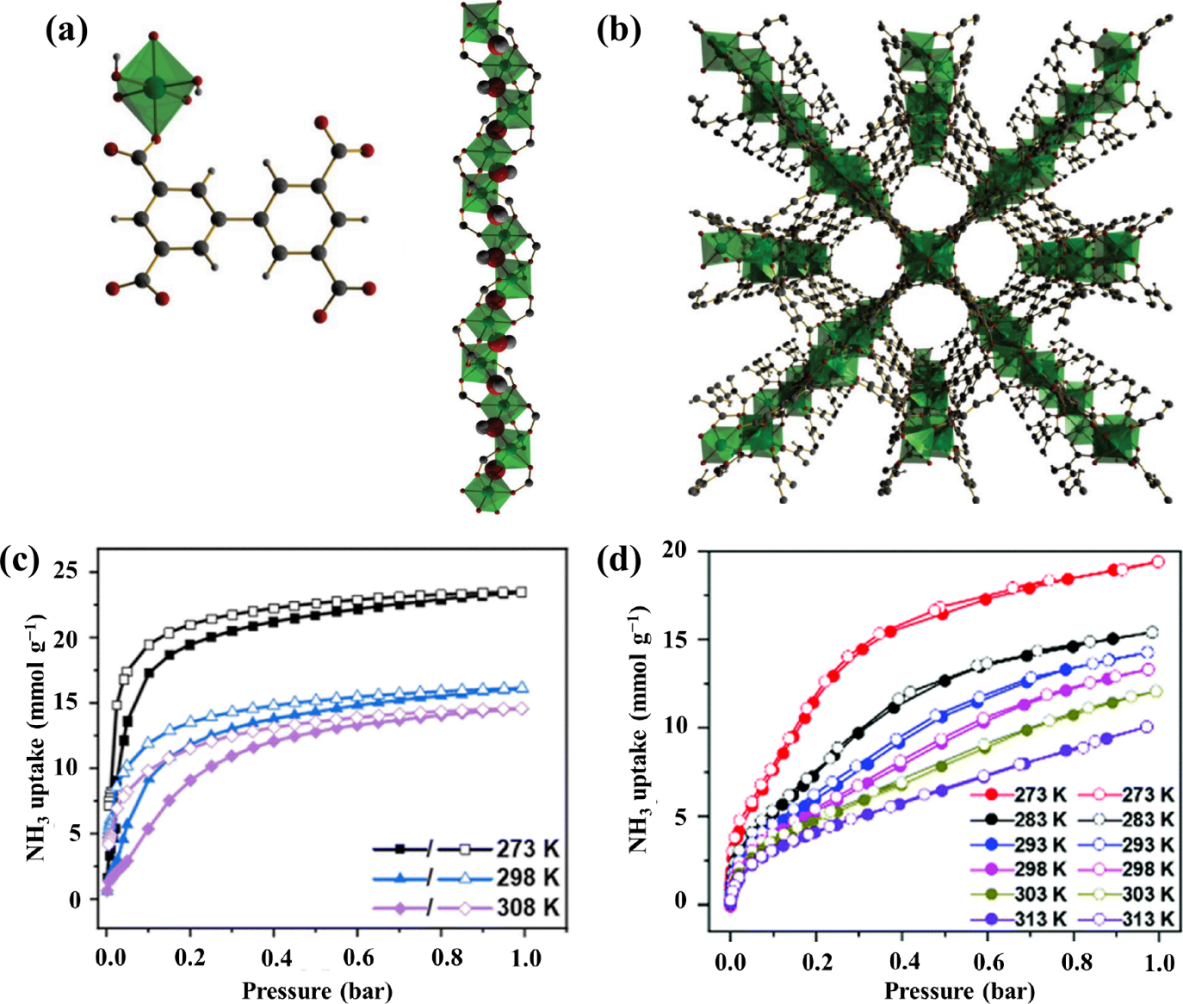
(a) View of the coordination environment of the ligand and metal center, and the corner-sharing extended octahedral chain of [MO_4_(OH)_2_]_∞_. The μ_2_-OH groups are highlighted as a space-filling model and are bound to the metal cation in a *cis* configuration. (b) View of the three-dimensional framework structure with channels formed along the *c*-axis. NH_3_ uptake isotherms of (c) MFM-300(Ti) and (d) MFM-300(Sc). Panels a and b are reproduced with permission from ref (41). Copyright 2012 Springer-Verlag. Panels c and d are reproduced with permission from refs (23) and (22). Copyright 2024 The Authors; copyright 2022 The Authors, respectively.

Higher valence metal ions (+3 or +4) usually form MOFs of higher stability, as they tend to form stronger metal–linker bonds. We have developed the MFM-300 series (MFM = Manchester Framework Material; M = Al^III^, Sc^III^, Fe^III^, Cr^III^, V^III^, V^IV^, In^III^, Ga^III^, and Ti^IV^) incorporating biphenyl-3,3′,5,5′-tetracarboxylate linkers for NH_3_ adsorption,^20−23^ having previously confirmed these to have high stability toward other corrosive gases such as SO_2_ and NO_2_.^41,42^ The framework consists of [MO_4_(μ_2_-OH)_2_] moieties {or [MO_4_(μ_2_–O)_2_] in the case of MFM-300(V^IV^) and MFM-300(Ti^IV^)} bridged by the linker to form a “wine-rack” structure with channels of approximately 6–8 Å width (Figure 2a,b). The hydroxyl groups {or bridging O^2–^ in the case of +4 metal ions} point into the pore. These materials do not incorporate vacant metal sites, and this likely enhances the stability of the resultant materials. Notably, adsorbed NH_3_ can form favorable supramolecular interactions particularly with the bridging hydroxyl or {μ_2_-O} groups and is thus held within the pores.

Adsorption capacities of 23.4 and 19.5 mmol g^–1^ at 273 K and 1 bar were found for MFM-300(Ti) and MFM-300(Sc), respectively (Figures 2c,d). Apart from the In and Ga analogues, all others in this series showed high stability toward NH_3_, with crystallinity retained after 20 cycles. As expected, higher BET surface area and pore volume lead to higher NH_3_ uptakes. During NH_3_ sorption, MFM-300(Ti) undergoes a structural change through elongation at the C–C bonds in the biphenyl linker likely attributed to the complex interlayer function of Ti^IV^ metal ions improving its NH_3_ uptake. The V^IV^ sites in MFM-300(V^IV^) is redox active, which results in a chemical conversion of NH_3_ on sorption to form reduced MFM-300(V^III^) and hydrazine (N_2_H_4_). We later showed that MFM-300(Fe) shows NH_3_ sensing capabilities, as it displays a high sensitivity toward NH_3_, attributed to the electron delocalization effects between the linker and Fe^III^ ions.^37^ This also confirms that the selective choice of metal ions may also introduce catalytic properties and functions into the framework.

In general, p-block metals often have comparatively weaker metal–oxygen bonds, making them less stable to NH_3_ which is reflected in the relative instability of MFM-300(In). Similar degradation of the framework structure has been observed for PMOFs composed of In^III^ and Ga^III^.^31^ In contrast, MFM-300(Al) displays remarkable stability toward NH_3_, retaining its structural integrity even after 50 cycles and was one of the first reported materials to display a liquid-like packing density of 0.60 g cm^–3^ at 298 K and 1 bar. Its long-term stability was also investigated, which confirms maintenance of structural integrity over four years.^35^ Comparing the ionic radii of Al^III^ and In^III^ (0.675 and 0.885 Å, respectively),^43^ it can be concluded that the higher charge density of Al^III^ results in exceptionally strong Al^III^–O bonds, improving the stability of the framework. Our work on MIL-160, a framework also composed of Al^III^ ions further supports this, showing remarkable stability with crystallinity of the framework being retained even after 16 pressure-swing cycles.^30^

Divalent metals have also been studied for NH_3_ sorption. The complexes [M_2_(DOBPDC)] (M = Mg^II^, Mn^II^, Co^II^, Ni^II^, Zn^II^, DOBPDC^4–^ = 4,4-dioxido-biphenyl-3,3-dicarboxylate) feature hexagonal channels with vacant metal sites.^26^ [Mg_2_(DOBPDC)] shows a high NH_3_ uptake capacity of 23.9 mmol g^–1^ at 298 K and 1 bar, with [Ni_2_(DOBPDC)] at 20.8 mmol g^–1^ reflecting the higher molecular weight of the latter. With the Lewis acidity of the exposed metal centers in the [M_2_(DOBPDC)] series being calculated as Ni > Mg > Co > Mn, further insight into the impact of Lewis acidity of the exposed metal centers is provided, with the Ni- and Mg-based frameworks exhibiting the highest NH_3_ adsorption capacities within the series. Notably, the structural integrity of all MOFs, apart from [Zn_2_(DOBPDC)], was retained upon cycling with NH_3_, confirming the recyclabilty of these materials.

The relationship of vacant metal sites to the stability of the framework and its uptake capacity can be rationalized in part by the Irving–William series, although thermodynamics and kinetics both play a role in observed NH_3_ uptake capacities and stability.^44,45^ The higher affinity of Cu^II^ toward NH_3_ is confirmed through both experimental and computational studies.^46−48^ Complexes of Cu^II^–NH_3_ are thermodynamically favored, but kinetically labile metal-linker bonds may dissociate upon contact with NH_3_. [Cu(CYHDC)] (CYHDC^2–^ = *trans*-1,4-cyclohexane-dicarboxylate) shows interconnecting one-dimensional chains of saturated [Cu_2_(OOCR)_4_] paddlewheel units and was found to adsorb NH_3_ through a step-shaped adsorption process at 20 mbar NH_3_, with an uptake of 17.5 mmol g^–1^ at 1 bar and 298 K.^28^ Upon exposure to NH_3_, a new one-dimensional nonporous polymeric phase, [Cu(NH_3_)_4_(CYHDC)], incorporating four equatorial NH_3_ and two CYHDC^2–^ ligands bound to Cu^II^ site was formed. Hysteresis upon isothermal desorption from this phase resulted in another intermediate phase, [Cu(NH_3_)_2_(CYHDC)] with the two NH_3_ molecules bound in the *trans* positions. The original [Cu(CYHDC)] phase can be regenerated. The interconversion between the original three-dimensional framework and the one-dimensional polymeric phases when exposed to NH_3_ is attributed to a ligand insertion mechanism, where one of the Cu–O_ligand_ bonds is cleaved to allow binding of NH_3_ to the Cu^II^ center. Interestingly, when 2,3,5,6-tetrafluoro-1,4-benzenedicarboxylate (TFBDC^2–^) and 4,4′-biphenyldicarboxylate (BPDC^2–^) linkers were used to synthesize isostructural frameworks, the initial adsorption step shifts to lower pressure in both cases, showing the effect of tuning the relatively labile Cu–O_ligand_ bond in this system.

[Fe(CYHDC)] exhibits a similar adsorption step but at a higher pressure of 70 mbar.^28^ Ni-based MOFs can show exceptional stability toward NH_3_ as exemplified above. However, they tend to show relatively low uptake capacities, with the exception of [Ni_2_(DOBPDC)], which may be related to the effect of the linker which contributes significantly to NH_3_ adsorption. The high stability of Ni^II^ materials is related to the thermodynamic contributions of high ligand field stabilization energies coupled with the relatively high Ni–linker dissociation energies.^49^

### 3.2 Ligand: Displacement and Functionalization

Modification of ligand structure and ligand functionalization are common approaches utilized within MOF chemistry to develop frameworks with improved properties and features, such as larger pore volumes and enhanced host–guest interactions, targeting better adsorption and storage. It is important that the bridging ligand forms strong, inert bonds with the metal ions for the generation of frameworks that are stable. If the metal–ligand bonds are weak and/or overly labile, NH_3_ adsorption may lead to framework collapse, as these bonds may be displaced with NH_3_, leading to permanent chemisorption. Ligand functionalization with polar or acidic functional groups serves as an important method to increase affinity toward NH_3_.

Hard Soft Acid Base (HSAB) theory offers alternative insights into the stability of MOFs toward NH_3_ and other corrosive gases.^50^ The MFM-300 series, consisting of metal ions in the +3 and +4 oxidation states, show exceptional stability toward most corrosive gases, which can be attributed to the strong metal–linker bonds and the absence of vacant metal sites. Metals of higher oxidation state are generally hard acids and thus form stronger metal–linker bonds with hard O-donors which are sufficiently inert to ligand displacement upon adsorption of NH_3_. Furthermore, linkers capable of forming stable chelating five- or six-membered rings show high stability toward strong basic solutions when bound to metal ions.^51^ Moreover, improving NH_3_ uptake at low pressure can be probed by introducing chemical functionalities to the linker to improve host–guest interactions. The linker geometry also allows control of the pore size of the material, allowing enhancement of both guest–guest and host–guest interactions, as NH_3_ molecules can be readily confined within the pore at low concentrations.

**Figure 3:**
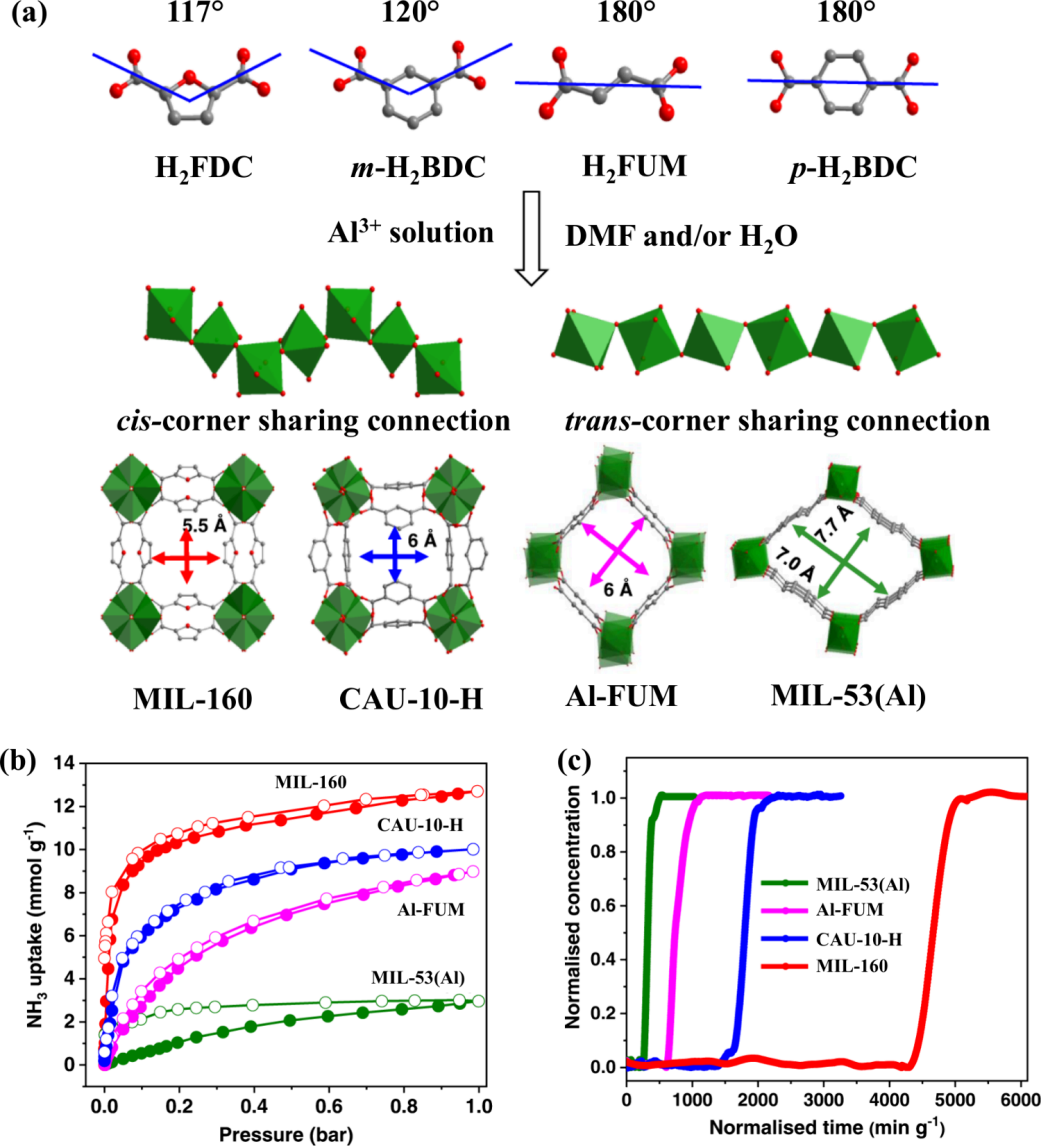
(a) Illustration of selected linkers and the self-assembly processes through *cis*- and/or *trans*-μ_2_-OH connected [AlO_6_] octahedral. (b) NH_3_ uptake isotherms at 298 K. (c) Dynamic breakthrough plots for NH_3_ (1000 ppm diluted in He) with an inlet gas flow rate of 25 mL min^–1^, showing a high NH_3_ retention for MIL-160. Figure reproduced with permission from ref (30). Copyright 2023 The Authors.

We have illustrated this concept through an investigation into NH_3_ sorption in a series of Al^III^-based MOFs, MIL-160, CAU-10-H, Al-FUM, and MIL-53.^30^ In all four materials, the Al^III^ centers are bound by two Brønsted acidic hydroxyl groups and four carboxylate ligands. MIL-160 and CAU-10-H feature 4-fold helical chains of corner-sharing [AlO_4_(OH)_2_] octahedra linked by *cis*-μ_2_-OH bridges and bent linkers FDC^2–^ (FDC^2–^ = furan-2,5-dicarboxylate) and *m*-BDC^2–^ (benzene-1,3-dicarboxylate), respectively. This creates “wine-rack” structures with square-shaped 1D channels (5–6 Å diameter) along the *c* axis. By using linear linkers, a different framework is formed in Al-FUM and MIL-53(Al), comprising *trans*-corner-sharing [AlO_6_] octahedra connected by FUM^2–^ (fumarate) and *p*-BDC^2–^ (benzene-1,4-dicarboxylate), resulting in 1D rhomb-shaped channels (Figure 3a).

Adsorption–desorption isotherms at 298 K and 0.001/1.0 bar show NH_3_ uptake of 4.8/12.8, 1.4/10.0, 0.47/9.0, and 0.07/3.0 mmol g^–1^ for MIL-160, CAU-10-H, Al-FUM, and MIL-53(Al), respectively (Figure 3b). Thus, an increase in surface area does not necessarily improve the NH_3_ uptake; the BET surface areas for MIL-160 and Al-FUM are 1000 and 1050 m^2^ g^–1^, respectively. The more accessible hydroxyl groups in the *cis* configurations improved the NH_3_ uptake in both MIL-160 and CAU-10-H, and the bent linker in MIL-160 provides abundant π-electrons, hydrogen-bonding capability, and narrow micropores which significantly improve the host–guest and guest–guest interactions through pore confinement. This improves the NH_3_ capture efficiency under a flow of 1000 ppm of NH_3_ diluted in He compared to other investigated materials (Figure 3c).

**Figure 4:**
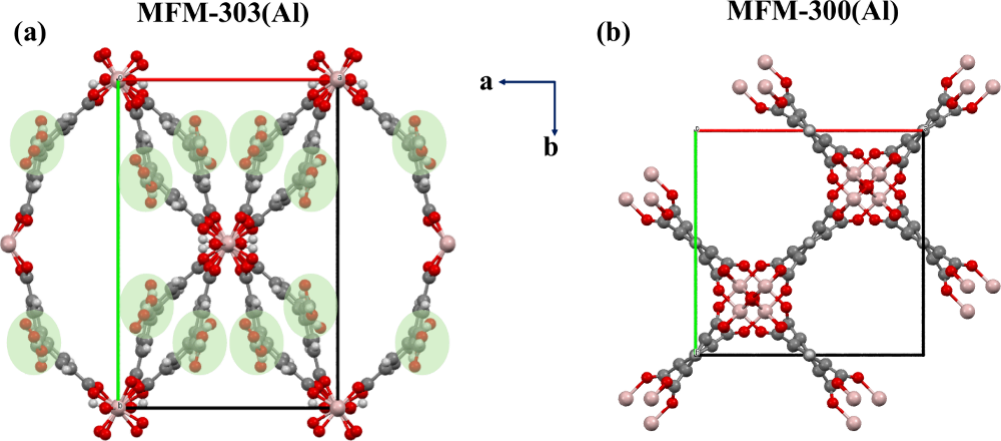
Views of (a) MFM-303(Al) and (b) MFM-300(Al) viewed along the *c* axis (pink = Al, red = O, black = C, white = H). The free COOH groups in MFM-303(Al) are indicated within the diagram.

Changes and even apparent minor alterations in the synthetic conditions used to prepare MOFs can lead to significantly different product(s) being isolated and can lead to *in situ* changes in pore decoration within the framework. For example, we reported MFM-303(Al),^24^ a highly acidic framework, which can be prepared *via* a modification of the synthesis of MFM-300(Al) using the same linker ligand. Addition of HCl to the reaction mixture used to prepare MFM-300(Al) results in the formation and isolation of MFM-303(Al). The addition of acid hinders the binding of the carboxylic acid group to Al^III^, resulting in the formation of a material incorporating free COOH groups (Figure 4). MFM-303(Al) shows a reversible NH_3_ uptake of 9.9 mmol g^–1^ at 273 K and 1 bar *via* hydrogen bonding of NH_3_ to the COOH group, resulting in a high packing density of 0.801 g cm^–3^ at 293 K. This is comparable to that of solid NH_3_ at 195 K (0.817 g cm^–3^).

A well-studied material for diverse functionalization is UiO-66, which is an ultrastable MOF composed of octahedral Zr(IV) metal clusters and 1,4-benzene-dicarboxylate (BDC^2–^). The breakthrough performance for dry NH_3_ (1000 ppm, 298 K) in UiO-66-NH_2_, incorporating pendant NH_2_ groups on the linker ligand, exceeds other derivatives incorporating acidic yet bulkier functional groups, such as COOH and SO_3_H. This was assigned to possible pore-clogging from the steric effect of larger moieties.^52^ Partial protonation of NH_2_ groups or reaction of NH_2_ groups with acetaldehyde to give hemiaminal or aziridine products lead to increased NH_3_ capacities.^53^ Despite the high stability of UiO-66 itself, these functionalized materials still reported a loss of uptake capacity, surface area, and crystallinity after long-term NH_3_ exposure during repeated cycling experiments. MOFs incorporating bipyridinium carboxylate linkers (viologens) such as 1,1′-bis(4-carboxyphenyl)-4,4′-bipyridinium and 4,4′-bipyridinium-1,1-bis(3-carboxyphenyl) also show improved NH_3_ uptake through strong donor–acceptor interactions between the linker and guest molecules.^54^

### 3.3 Secondary Building Units: Defects and Decoration

Altering the NH_3_ adsorption performance through modification of secondary building units has also gained increasing interest. Formation of defect sites in both UiO-66 and UiO-67 through the removal of carboxylate linkers from within the structure allows *in situ* binding of metal ions to enhance gas adsorption properties. This can be achieved *viain situ* solvothermal synthesis and by use of plasma technology. For example, doping of the OH site in UiO-66-defect with Cu^II^ ions increases NH_3_ uptake capacity to 16.9 mmol g^–1^ at 273 K and 1 bar, with the parent material showing an uptake of 11.8 mmol g^–1^ under the same conditions.^29^ This 43% increase in uptake is explained by the presence of additional vacant metal sites within the Cu^II^-functionalized framework. Interestingly, this framework shows high stability toward pressure-swing NH_3_ adsorption, with crystallinity being retained after 15 cycles. As previously stated, most frameworks based upon M^2+^ metal centers tend to collapse on NH_3_ adsorption, but the Zr_6_ clusters based upon Zr^IV^ centers bound to the carboxylic linkers ensure that structural stability is retained. We later demonstrated that incorporation of Cu^II^ sites into the framework improves the electrocatalytic conversion of nitrates to NH_3_.^36^

Treatment of the Zr_6_ cluster in NU-1000 with HCl leads to enhancement of both reversible and irreversible uptake of NH_3_ compared to the parent material. Formation of NH_4_^+^ cations upon NH_3_ adsorption *via* reaction with H_2_O ligands at the cluster node was observed in both chlorinated and nonchlorinated materials, with irreversible NH_3_ adsorption observed on chlorinated sites. Furthermore, the higher uptake in NU-1000-Cl was facilitated through the presence of Cl^–^ ions to form halogen–hydrogen interactions.^55,56^ Doping LiCl (43.4 wt%) into MIL-53-(OH)_2_ affords a material that shows exceptionally high NH_3_ uptake capacity of 33.9 mmol g^–1^ at ambient conditions due to the synergistic interaction between NH_3_ and Li^+^ ions confined within the nanopores and due to the presence of halogen–hydrogen bonding between NH_3_ and the Cl^–^ ions.^25^

Selection of a stable parent material and improvement of the NH_3_ affinity and capacity through ligand derivatization are widely applied strategies. However, postsynthetic modification of linker ligands remains challenging considering potential thermodynamic and kinetic barriers to the formation of new bonds on a linker within a framework. Recently, through consecutive postsynthetic modification with acidic functional groups in the mesoporous material [Ni_2_Cl_2_(BTDD)] (BTDD^2–^ = bis(1*H*-1,2,3-triazolato[4,5-*b*],[4′,5′-*i*])dibenzo[1,4]dioxin), the material Ni_acryl_TMA (TMA^2–^ = thiomallate) has been fabricated.^27^ Exchange reactions of bridging ligands at room temperature on [Ni_2_Cl_2_(BTDD)] afforded Ni_acryl, which was then transformed *via* a photocatalytic enol–thiol click reaction to form Ni_acryl_TMA. Significantly, photocatalytic reactions are often milder than most other strategies for ligand functionalization and derivatization, and this allows retention of the original framework structure. N_2_ isotherms at 77 K showed a reduced pore volume on functionalization, with the BET surface area dropping from 1762 m^2^ g^–1^ to 1128 m^2^ g^–1^ for Ni_acryl_TMA. However, at 298 K and 1 bar, Ni_acryl_TMA exhibits a high isothermal NH_3_ uptake of 23.5 mmol g^–1^. The recyclability and stability of this polyfunctionalized material are demonstrated in cyclic adsorption–desorption experiments with only minor loss of capacity.^27^

## 4 Characterizing Ammonia Adsorption within MOFs

Most MOFs that show promising performance for reversible NH_3_ adsorption and capture fall in the microporous range. Therefore, direct host–guest interactions between the framework and NH_3_ significantly affect and control the adsorption performance, capacity, and characteristics of the material. Different types of host–guest interactions between MOFs and adsorbates include coordination, hydrogen, and halogen bonds as well as electrostatic interactions.^57^ Specifically, with NH_3_ as guest molecules, the most common host–guest interactions are direct coordination bonds to vacant metal sites and hydrogen bonding or electrostatic interactions with functional groups on the organic linker. The study of such interactions in these systems, however, remains challenging even though MOFs often showing high crystallinity and ordered structures. Synthetic modifications may increase further the complexity of the resultant host material. Neutron powder diffraction (NPD), inelastic neutron scattering (INS), *in situ* infrared ( IR) microspectroscopy and electron paramagnetic resonance (EPR) and solid-state NMR (ssNMR) spectroscopy have been used to elucidate the mechanisms of binding of NH_3_ and associated substrates within MOFs, and we discuss these techniques below.

**Figure 5:**
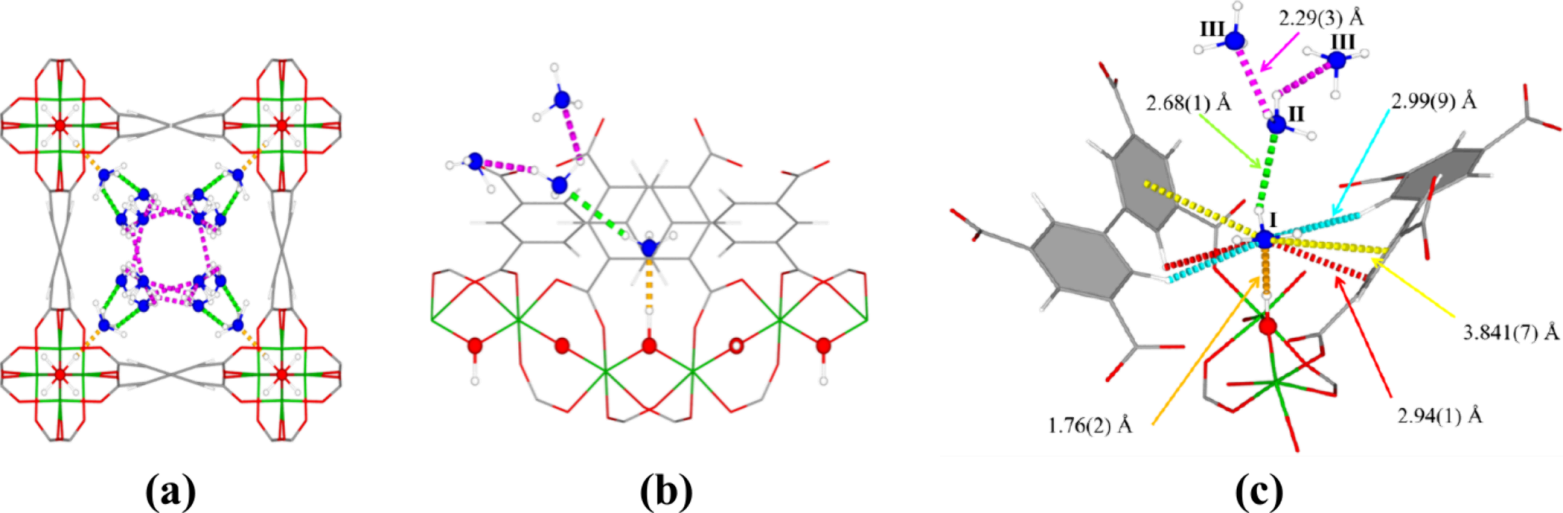
Views of NH_3_-loaded MFM-300(Al) (loading 1.5 ND_3_/Al) determined by *in situ* neutron powder diffraction: (a) packing of NH_3_ along the pore; (b, c) host–guest interactions between ND_3_ and the framework. Figure reproduced with permission from ref (21). Copyright 2018 The Authors.

Visualization of the host–guest and guest–guest interactions between NH_3_ and the framework is crucial to understand how these materials function and to improve these properties further. In our studies, we have utilized NPD, which is highly sensitive to light atoms and is able to distinguish between H and D, to afford a more concise and clear comparison between desolvated and ND_3_-loaded frameworks. For example, we have investigated the binding sites of ND_3_@MFM-300(Al) using NPD (Figure 5) which confirms that the primary binding site [site I, occupancy = 0.736(6)] involves formation of a hydrogen bond with the bridging μ_2_-OH group. This is accompanied by further hydrogen bonding and electrostatic interactions with the aromatic rings of the linker.^21^ Reversible H–D exchange is also observed at higher loading between μ_2_-OH and loaded ND_3_. Site II [occupancy = 0.236(3)] and site III [occupancy = 0.213(5)] are based upon guest–guest interactions, forming a cooperative hydrogen bonding network that propagates along the pore.

**Figure 6:**
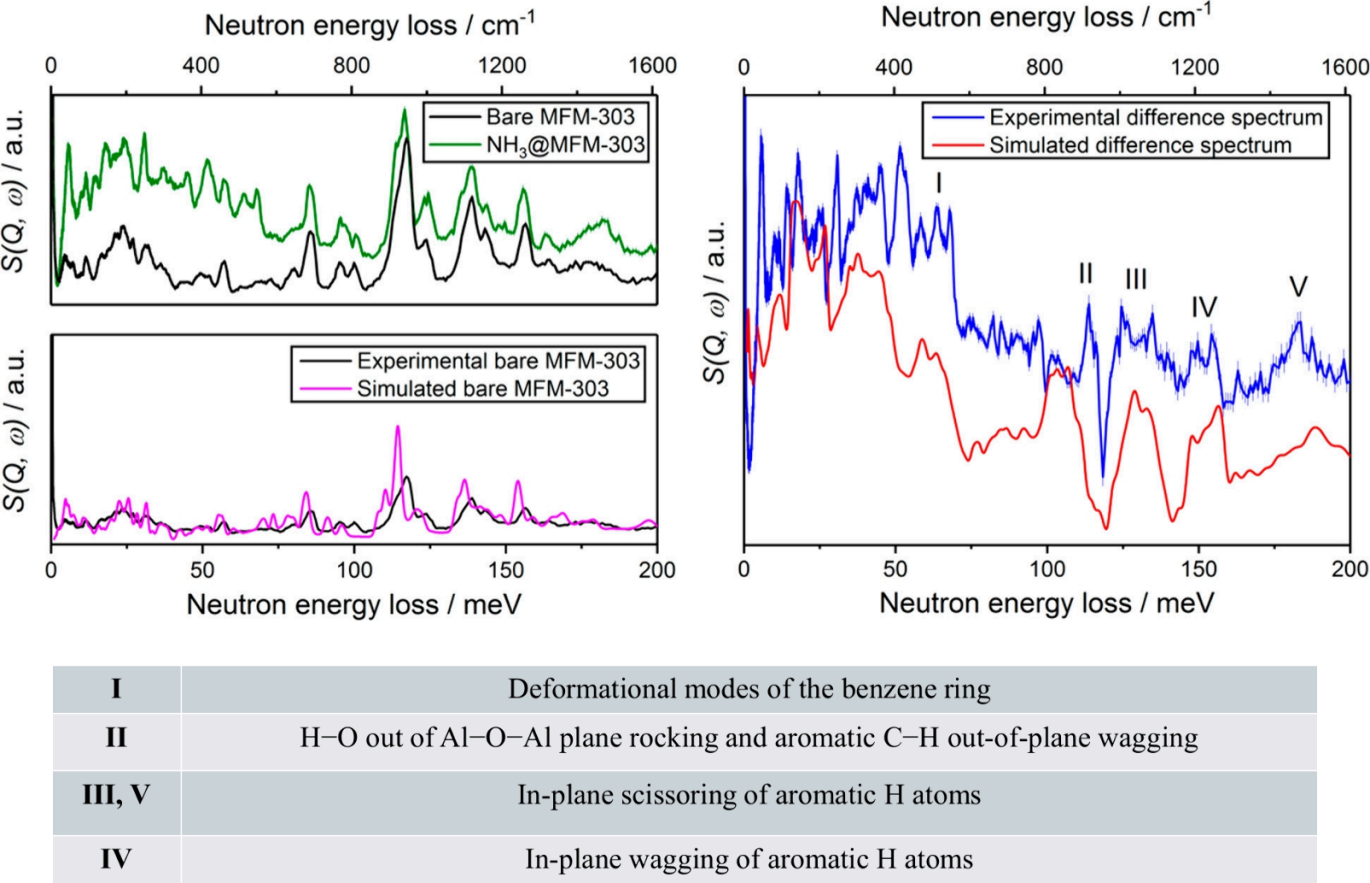
Experimental and simulated inelastic neutron scattering spectra of bare and NH_3_-loaded MFM-303(Al) with peak assignments confirmed by DFT calculations. Figure reproduced with permission from ref (24). Copyright 2021 The Authors.

To gain further insights into the dynamic behavior of NH_3_ storage in MOFs, INS is a promising technique. INS is sensitive to hydrogen-based vibrations, is independent of optical selection rules, and offers information over a broad energy range from 80 to 4000 cm^–1^. For MFM-303(Al), a stable framework featuring free pendant carboxylic acid groups and available hydroxyl sites, exceptional NH_3_ packing density was achieved (0.801 g cm^–3^).^24^ By combination of simulation of *in situ* INS and DFT data, each vibrational mode across the energy loss region can be assigned, as shown in Figure 6. Peaks in the higher energy region in the difference spectra reflect the changes in hydrogen-based vibrational modes of the framework upon adsorption of NH_3_. Interactions between guest NH_3_ molecules and the free carboxylic acid and bridging hydroxyl groups as well as with aromatic rings are reflected in increases in the peak intensity and shifts of peak positions.

**Figure 7:**
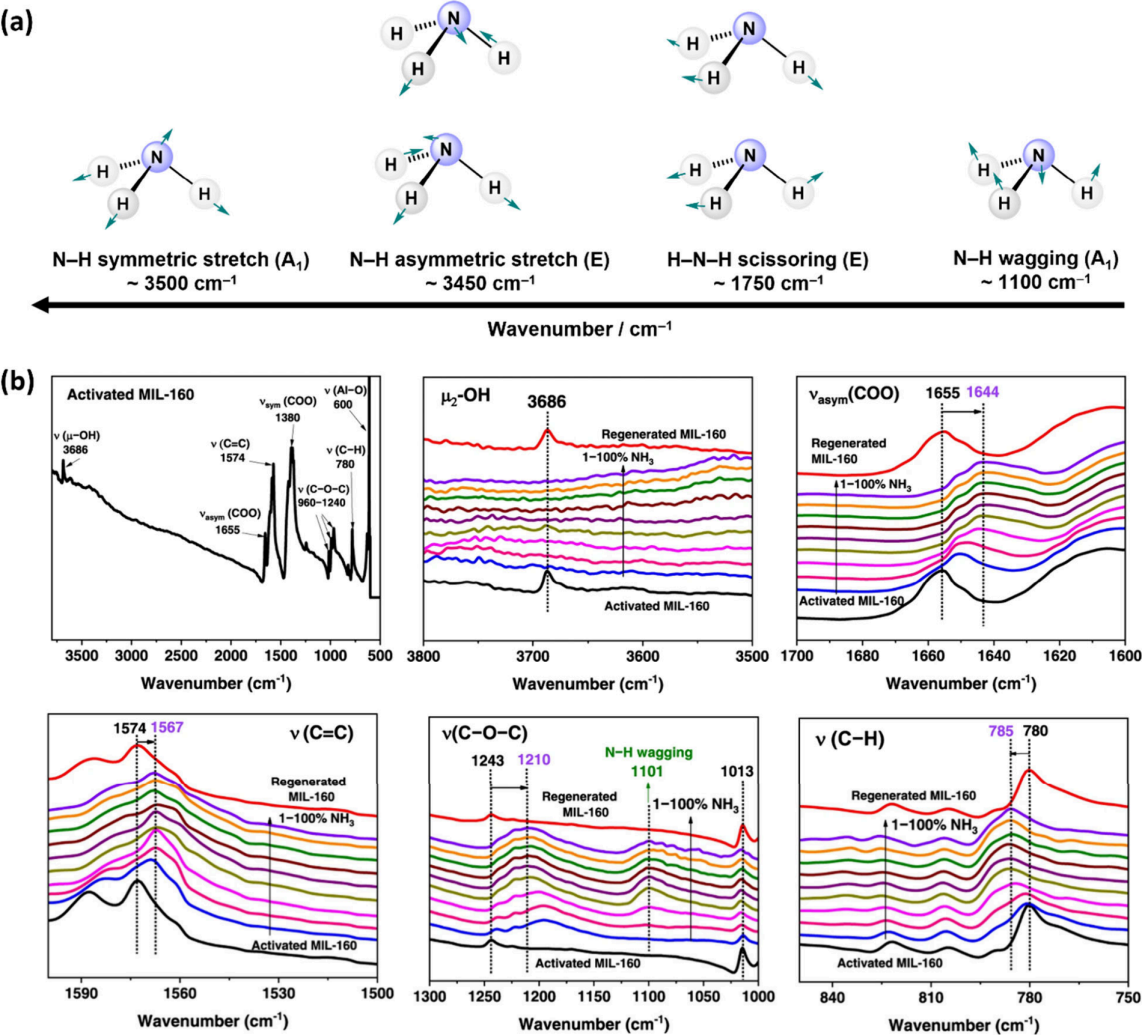
(a) Vibrational modes of NH_3_. (b) *In situ* synchrotron IR spectra for pristine and NH_3_-loaded MIL-160 (NH_3_ diluted in dry N_2_). Panel b reproduced with permission from ref (30). Copyright 2023 The Authors.

NH_3_ has four active vibrational modes (Γ_vib_ = 2A_1_ + 2E) (Figure 7a), allowing detection and measurement of adsorbed NH_3_ within the pore using IR spectroscopy. *In situ* IR microspectroscopy also allows interactions between NH_3_ and the framework to be observed. In MIL-160 a decrease in intensity of the O–H stretching band at 3686 cm^–1^ was observed upon loading with NH_3_ when a flow of 1% NH_3_ was introduced.^30^ This is consistent with the primary binding site determined by neutron powder diffraction (NPD) studies. Interaction between NH_3_ and linker is shown by a gradual red shift of carboxylate group asymmetric stretching (1655 cm^–1^), C=C stretching (1574 cm^–1^), and a blue shift of the C–H deformation band (785 cm^–1^) (Figure 7b). Thus, the importance of the softer interactions between NH_3_ and the linker aryl groups are highlighted. In MFM-300(Ti), we also observe a notable change in the C–C vibration mode, where the bands at 1548 and 1500 cm^–1^ merge to a single band at 1525 cm^–1^ when the NH_3_ flow was increased from 2% to 5%, suggesting a change in the conjugated structure of the aromatic rings.^23^ This observation was also supported by refinements obtained from NPD which showed an elongation of the C–C bonds on increasing ND_3_ loading.

Several studies have also utilized magnetic spectroscopic techniques to identify possible structural changes upon NH_3_ adsorption and to give further understanding and insights into the nature of binding interactions. The host–guest charge transfer in MFM-300(V^IV^) during NH_3_ adsorption was studied using EPR specroscopy.^20^ Activated MFM-300(V^IV^) shows a single resonance peak at *g* = 1.955 at X-band frequency (9.86 GHz) that disappeared upon loading of NH_3_, with the 3d^1^ V^IV^ metal center being reduced to V^III^*via* a single electron charge-transfer process with concomitant formation of hydrazine H_2_N–NH_2_. ssNMR spectroscopy has also been applied to the study of NH_3_ interactions within MOFs. In NH_3_-loaded UiO-66-defect, ^1^H magic angle spinning (MAS) ssNMR shows a large narrow signal from NH_3_ at δ{^1^H} = 2.8 ppm, with a full width at half-maximum (FWHM) of approximately 650 Hz.^29^ The same measurements on NH_3_-loaded UiO-66-Cu^I^ and UiO-66-Cu^II^ did not show this large peak, indicating that NH_3_ molecules adsorbed were more confined within the pore, whereas in NH_3_-loaded UiO-66-defect, rapid free motion of NH_3_ was possible thus generating the observed signal. Confinement of NH_3_ within the pores causes a reduction in the degrees of freedom in UiO-66-Cu^I^ and UiO-66-Cu^II^ consistent with direct coordination of NH_3_ to vacant metal sites, again consistent with the ssNMR spectroscopy. Dipolar interactions can also be determined by ssNMR specroscopy. For example, ^1^H–^45^Sc heteronuclear dipolar correlation spectroscopy (HETCOR) MAS ssNMR was used to study NH_3_-loaded MFM-300(Sc) to confirm hydrogen bonding interactions between the bridging hydroxyl (μ_2_-OH) groups and the NH_3_ molecules within the pore.^22^

## 5 Conclusion and Outlook

Increasing global demand for NH_3_ and increasing levels of pollution call for cheaper, less energy-intensive, efficient storage and capture processes. MOFs have been extensively studied over the past few decades for a wide range of substrate separations and conversions. However, as noted in this Account, there remain very few stable porous MOFs that show high uptakes and high stability over multiple cycles toward this highly corrosive substrate. It is worth noting that although NH_3_ storage in MOFs has formally achieved the so-called US DoE ultimate target in terms of H_2_ storage, its subsequent conversion to H_2_ remains a future target. This Account offers a systematic understanding of the methods reported to control, enhance, and study NH_3_ uptake and improve stability based on design and selection of host–guest interactions within porous framework hosts. With the very high tunability and design of MOFs available by choice of metal coordination and cluster nodes and of organic linkers and postsynthetic methodologies, the collective impact of surface functionalities (vacant metals sites and functional groups), pore size and volume, and structural stability have significant impact on reversible NH_3_ adsorption performance. The direct understanding and visualization of host–guest interactions in these relatively complex systems are vital for future development of new materials, which calls for advanced characterization techniques, including advanced diffraction, scattering, and other spectroscopic and dynamic methods. This Account summarizes the current state-of-the-art for NH_3_ storage by MOFs and reflects upon the future development and design of new and better materials for NH_3_ adsorption, separation, and conversion, an area that remains in its infancy.
